# Combined network analysis and interpretable machine learning reveals the environmental adaptations of more than 10,000 ruminant microbial genomes

**DOI:** 10.3389/fmicb.2023.1147007

**Published:** 2023-09-20

**Authors:** Yueyang Yan, Tao Shi, Xin Bao, Yunpeng Gai, Xingxing Liang, Yu Jiang, Qiushi Li

**Affiliations:** ^1^Key Laboratory for Zoonoses Research of the Ministry of Education, Institute of Zoonosis, College of Veterinary Medicine, Jilin University, Changchun, China; ^2^College of Animal Science and Technology, Northwest A&F University, Yangling, China; ^3^Department of Stomatology, Taian Central Hospital, Tai'an, Shandong, China; ^4^School of Grassland Science, Beijing Forestry University, Beijing, China; ^5^Department of Stomatology, The Fifth Affiliated Hospital of Sun Yat-sen University, Zhuhai, Guangdong, China

**Keywords:** ruminants, metagenomics, machine learning, network, metagenome-assembled genome

## Abstract

**Background:**

The ruminant gastrointestinal contains numerous microbiomes that serve a crucial role in sustaining the host’s productivity and health. In recent times, numerous studies have revealed that variations in influencing factors, including the environment, diet, and host, contribute to the shaping of gastrointestinal microbial adaptation to specific states. Therefore, understanding how host and environmental factors affect gastrointestinal microbes will help to improve the sustainability of ruminant production systems.

**Results:**

Based on a graphical analysis perspective, this study elucidates the microbial topology and robustness of the gastrointestinal of different ruminant species, showing that the microbial network is more resistant to random attacks. The risk of transmission of high-risk metagenome-assembled genome (MAG) was also demonstrated based on a large-scale survey of the distribution of antibiotic resistance genes (ARG) in the microbiota of most types of ecosystems. In addition, an interpretable machine learning framework was developed to study the complex, high-dimensional data of the gastrointestinal microbial genome. The evolution of gastrointestinal microbial adaptations to the environment in ruminants were analyzed and the adaptability changes of microorganisms to different altitudes were identified, including microbial transcriptional repair.

**Conclusion:**

Our findings indicate that the environment has an impact on the functional features of microbiomes in ruminant. The findings provide a new insight for the future development of microbial resources for the sustainable development in agriculture.

## 1. Introduction

Ruminants, as ancient animals, exhibit a wide range of morphological and ecological diversity (Mennecart et al., 2021). They have adapted to diverse habitats, from tropical jungles (Díaz-Céspedes et al., 2021) to the plateau (Guo et al., 2020); range in size from 2 kg (Pickford, 2001) to 1.5 tons (Sauer et al., 2016); show great variations in diet, feeding on objects ranging from moss (Ihl and Barboza, 2007) to ordinary standard feed (Li et al., 2019); and have adapted to almost all ecosystems on Earth. Ruminants are distinguished by their plant digestion patterns and have evolved the rumen. As one of the most vital organs, the rumen allows partial microbial digestion of feed before it enters the true stomach (van Lingen et al., 2017). The rumen is a crucial factor underlying the domestication of ruminants. The productivity of ruminant livestock depends on their gastrointestinal microbiota, which can transform plant material that humans cannot digest into easily accessible animal products (Shabat et al., 2016). The gastrointestinal microbiota of ruminants is characterized by its diversity and dynamic nature, making it prone to alterations due to changes in diet (Malmuthuge and Guan, 2016), environmental factors (Cholewinska et al., 2021), and the presence of enteric pathogens (Cortés et al., 2020). These perturbations play an integral role in host nutritional intake, behavior, metabolism, immunological function, and development. Natural selection has allowed hosts and symbiotic microbes to evolve as integrated systems.

In both the ecological and social realms, ruminants are immensely valuable. Due to rising consumer demand for animal products resulting from population growth (Seshadri et al., 2018), ruminants play an increasingly vital role in ensuring agricultural security. They generate a significant amount of the meat and milk that are the primary sources of protein in the human diet (Stewart et al., 2019). Nonetheless, sustainable manufacturing confronts significant obstacles due to the depletion of natural resources and the resulting rise in production costs.

The ability of ruminants to utilize microorganisms is one of their key traits. Microorganisms bring significant benefits to ruminant animals. However, due to the diverse functionalities and species diversity of microorganisms, they exhibit intricate physiological and biochemical characteristics, making their in-depth analysis quite challenging (Ban and Guan, 2021). We have uncovered inconsistencies in predicting microbial community structures, primarily stemming from a limited grasp of the mechanisms governing microbial community assembly. In order to mitigate this unpredictability, it is imperative to comprehensively understand the microbiome as a cohesive entity.

With the ongoing increase in the depth of metagenomic sequencing, the range of sequencing is progressively expanding (Vestergaard et al., 2017), while the cost of the technology is decreasing. Thus, large amounts of data can be generated for analysis. Consequently, substantial volumes of data can be generated for analysis. However, despite genomics being inherently data-driven, the resultant datasets are becoming both exceedingly large and complex, thereby giving rise to technological challenges. Recent publication of the most recent collection of gut microbial genomes includes a ruminant whole gastrointestinal tract microbial gene set and the reconstruction of over 10,000 nonredundant ruminant gastrointestinal microbial genomes (Xie et al., 2021). This represents a significant change in the ability to understand the ruminant microbiome. This study used public database collections to characterize the microbiomes and functional groups and applied a metagenomics approach to achieve the following objectives: (1) building microbial cooccurrence networks for exploring linkages in microbial communities, (2) the influence of the network’s special microstructure on the survivability of gastrointestinal networks in ruminant was explored, (3) detecting antibiotic resistance in different ruminants on a large scale, and (4) exploring the adaptation of ruminant microbes to their environment.

## 2. Results

### 2.1. Microbiological characteristics of the gastrointestinal microbiota of ruminants

**Figure 1:**
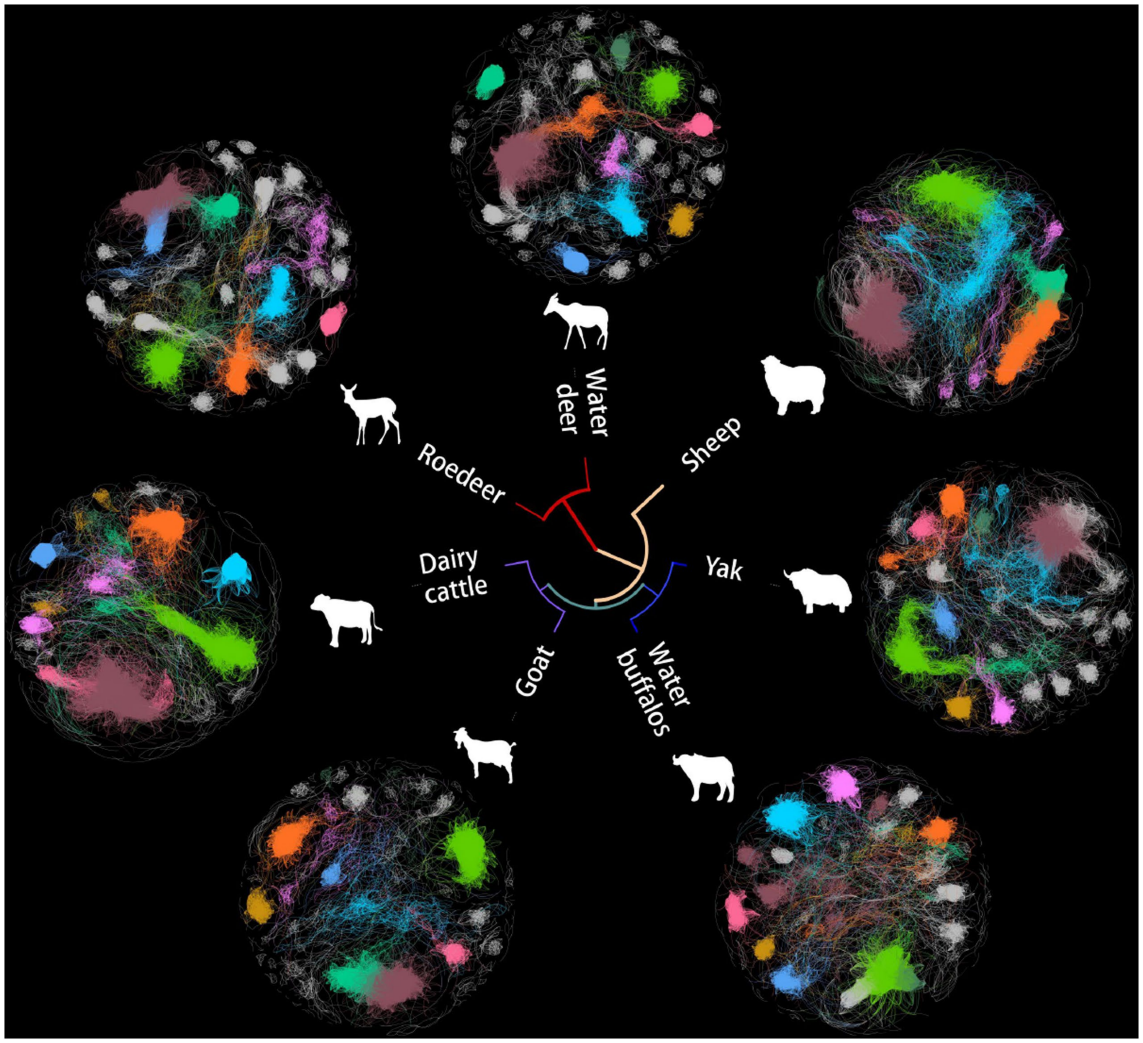
Networks of co-occurrence among seven ruminant microbes. Unrooted tree exhibiting a Bray-Curtis clustering tree of microbial genera. The first eight domain modules in the cooccurrence network are displayed in various colors in the outer circle, which displays various animal microbial cooccurrence networks.

The microbes serve as an often overlooked yet independent source of data for understanding host evolution and ecological shifts. Based on Spearman’s rank correlation coefficient matrix of the relative abundance of 488 collected microorganisms, a microbial network was constructed using seven microbiomes representing distinct ruminant gastrointestinal environments (Figure 1). To eliminate noise and false positive predictions, a conservative statistical cutoff was adopted to reject points with Spearman coefficients less than 0.85 and *p-*values less than 0.01 The number of modules in this global network was distributed with a long tail, with an average of 32 modules per network, while 80% of the vertices were concentrated in only 8 modules.

**Figure 2:**
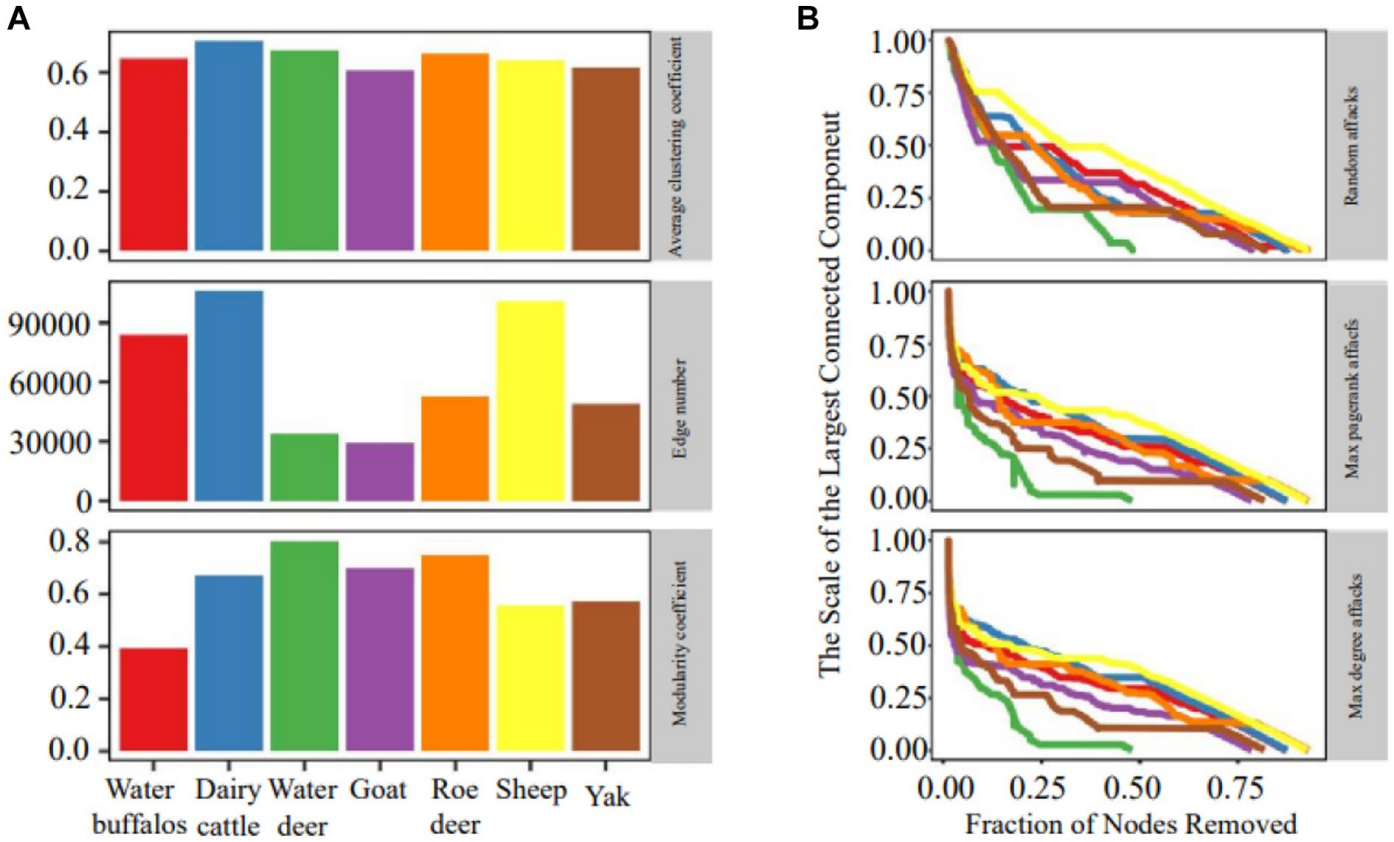
Network topology and robustness. **(A)** Microbial network topology inferred from a dataset of microbiome abundance of seven ruminant species. **(B)** The relationship between the proportion of removed nodes and the scale of the largest connected component.

The organization of ruminant gastrointestinal microbial networks varies greatly, and there is minimal relationship between the various species (Figure 2A). Dairy cattle had the highest mean clustering coefficients, which indicated substantial clustering and indicated that the network’s nodes tended to form relationships over shorter distances. Sheep, dairy cattle and water buffaloes all had more edges than the others. The quantity of edges varied notably among networks, with dairy cattle networks exhibiting three times the number of edges as compared to goat networks. Additionally, we found that all networks had a modular structure (modularity > 0.3) and dense connections between nodes within modules.

Figure 2B demonstrates that the random attack is less effective than the purposeful approach, showing that the microbial network is more resistant to random attacks. During simulations of purposeful attacks, the network stability rapidly degrades and shows a clear declining trend, illustrating the superiority of the DG (max degree graph node) and PG (max pagerank graph node) importance ranking-based techniques. Sheep had the most resilient microbial network regardless of the technique of hitting, while water deer and yak networks had the most susceptible microbial networks. The efficiency of network attacks using the same method varied in a multimethod robustness assessment of individual networks, with PG attacks performing better in elk and less successfully in deer.

### 2.2. The K-shell decomposition in gastrointestinal microbiota of ruminants

**Figure 3:**
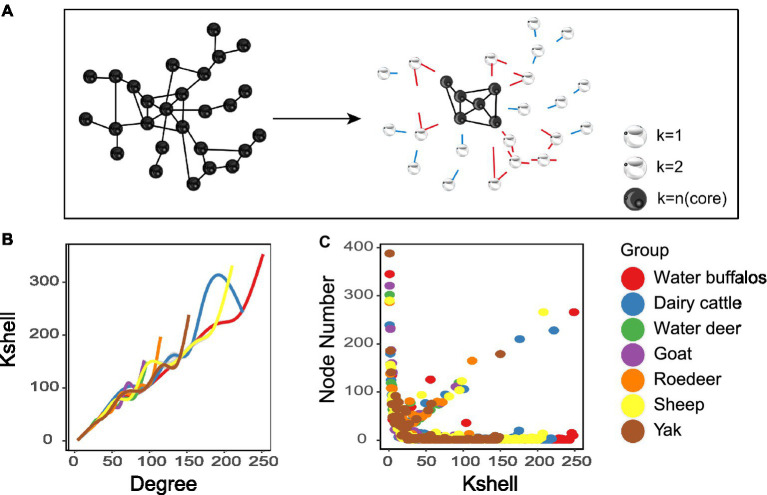
Network analyses of K-shell. **(A)** Schematic illustration of network layering using the k-shell decomposition method. **(B)** Distribution of the average degree of each shell. **(C)** each shell number.

The K-shell technique is utilized to examine the network’s hierarchical structure. The procedure is run repeatedly until the most concentrated core is discovered (Figure 3A). Nodes dispersed at the network’s periphery have a much lower average than nodes positioned close to its core (Figure 3B). The statistics suggest that inner layer nodes may be more efficient information emitters (Figure 3C). We also found that the overall structure of the microbial network topology is reminiscent of the internet network. While merely 0.5% of the nodes in the internet network constitute the nucleus, a mere 0.1% of the nodes comprise the core of the microbiological network.

## 3. Broad-spectrum profile of ARG abundance in ruminant gastrointestinal

**Figure 4:**
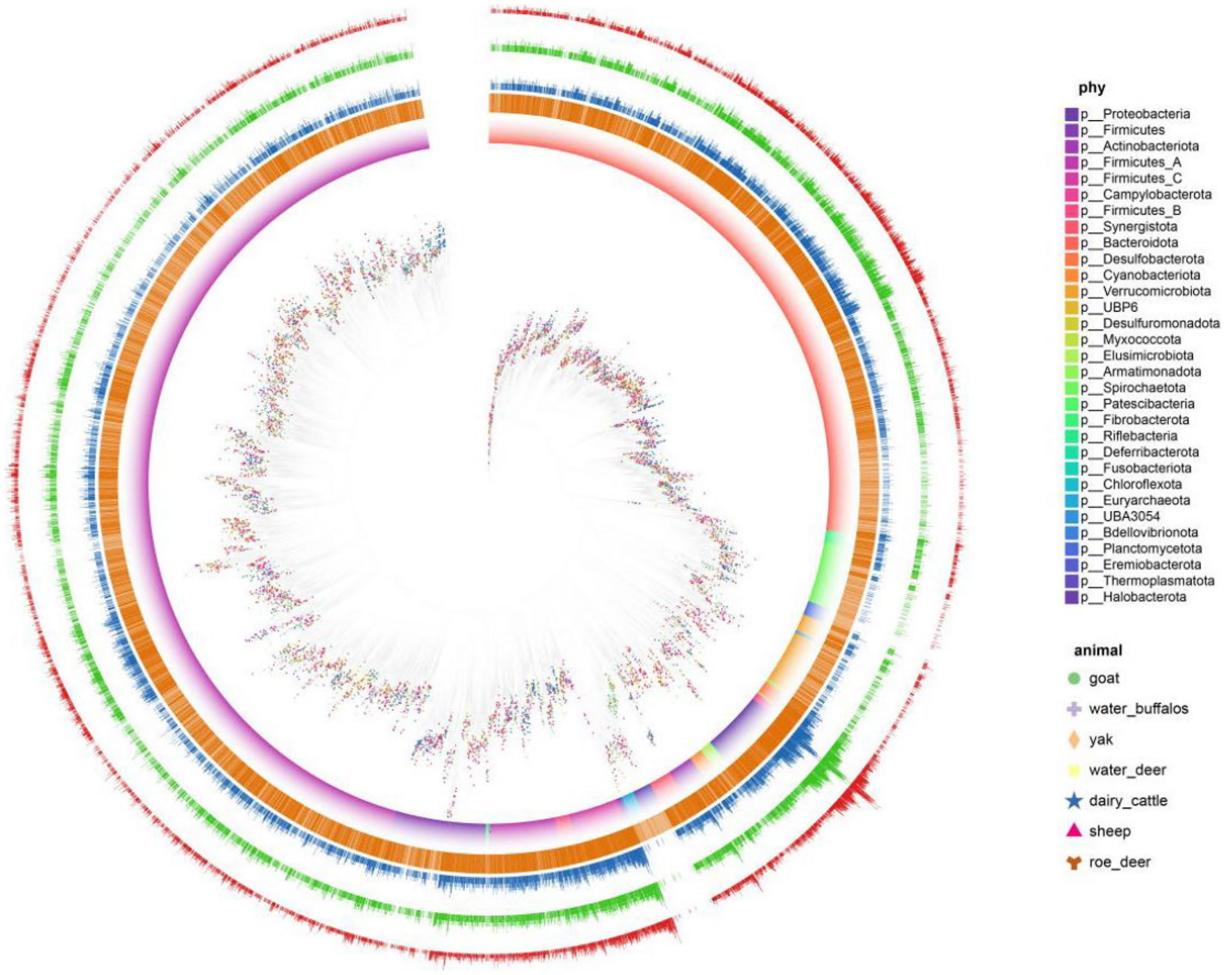
Antibiotic-resistance distribution in gastrointestinal microbes. Clades are 146 colored according to phyla and SHAP represents different hosts. The first layer uses 147 orange represents the genome of antibiotics. Second, three, and the four layers represent 148 the number of ARG, how many types and the ARG risk index.

Antibiotics stand as influential elements in microbial networks, exerting substantial impacts. Unveiling antibiotic resistance genes’ presence holds the potential to influence the operational dynamics of microbial communities and the overall stability of ecosystems. We discovered a significant prevalence of antibiotic resistance genes (ARGs) in ruminant gastrointestinal microbes, with 6,268 (62%) of the 10,073 genomes tested in the microbial resistance gene analysis yielding positive results. Multiple ARGs demonstrated a high degree of variability within the ruminant resistance group (Figure 4). We also quantified the possible mechanisms of the identified ARGs. Multiple ARGs demonstrated a high degree of variability within the ruminant resistance group. We found that yaks had the fewest ARGs, with an average of 181 ARGs per sample. We hypothesize that this is because the Qinghai–Tibet Plateau is less contaminated as a result of human activities.

We further analyzed the detailed composition of ARGs in the gastrointestinal of seven ruminants. Among all the types of ARGs, uppP, tufab, tetW, tetT, tet37, rpsL, rpsJ, rpoC, rpoB, parE, nimJ, macB, and gyrAAPH (2″)-Ig, which had the highest prevalence, were found in the gastrointestinal tracts of all ruminants. We quantified the risk index of species containing risk genes using quantitative methods, and we characterized the risk level of MAG based on the risk score quartile. The risk level is divided down into 4 levels: 2611 (risk index 1), 1,491 (1 = risk index 10), 657 (10 = risk index 100), and 62 (risk index ≥100). The total number of level 1 microorganisms was 62, or 1.2%. We displayed the tick microbial symbiont risk indicator top 50 in. We discovered that among the top 10 MAGs of all hazards, there were members of Pseudomonas, Escherichia, and Acinetobacter.

Explanation of the machine learning-based analysis of the evolution of gastrointestinal microbial environmental suitability in ruminants.

**Figure 5:**
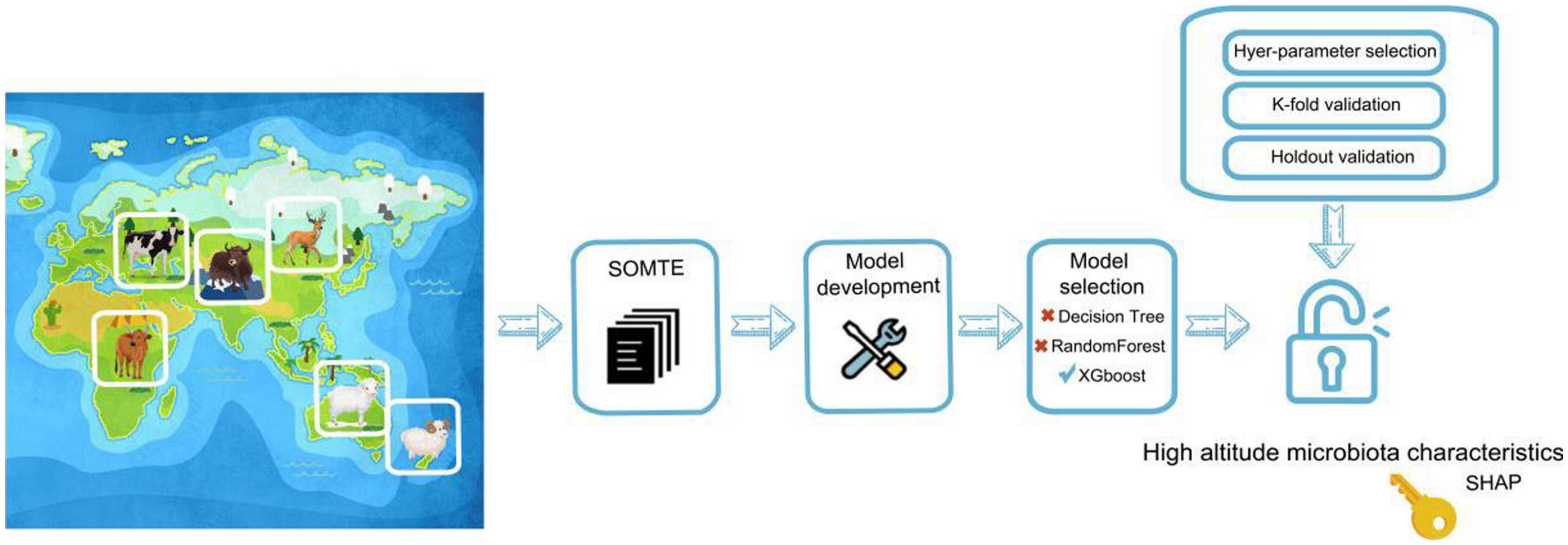
Flow chart of machine learning implementation for predicting the adaptive function of microorganisms.

Many creative strategies are supported by machine learning that can detect patterns and trends in huge data that cannot be identified using traditional analysis-based methods. To this end, we further investigated the differences between rumen microbes at different altitudes using the Kyoto Encyclopedia of Genes and Genomes (KEGG) database based on feature engineering. The 330 yak rumen microbial genomes were subjected to KEGG annotation with 1992 other ruminant rumen microbial genomes to generate a KEGG microbial function matrix (Figure 5) that indicated whether each genomic source was from a high altitude. Thus, the raw data were transformed into features that were more representative of potential problems with the prediction model. The ratio of the number of high-and low-elevation samples was approximately 1: 6. The significant imbalance between these two categories was evident. In order to balance the two sets of sample data, our study employed the synthetic minority oversampling technique (SMOTE) algorithm, which is a completely sampled synthetic data algorithm.

**Figure 6:**
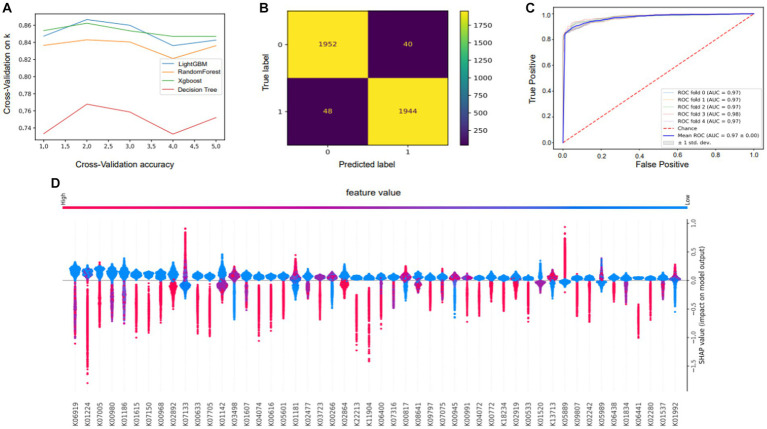
Performance and characteristics of the machine learning model. **(A)** Selection of model algorithms based on 10 cross-validation sets: lightGBM is the blue line, XGBoost is the green line, random forest is the yellow line and the decision tree is the red line. **(B)** Confusion matrix showing the performance of the LightGBM model. **(C)** Receiver operating characteristic (ROC) curve of the RF model. The horizontal coordinate of this curve is the false-positive rate (FPR), and the vertical coordinate is the true-positive rate (TPR). **(D)** Plots summarizing the SHAP values of all the samples were used for analysis to interpret key features.

We developed four models of machine learning. The accuracy and applicability of the various techniques were assessed using cross-validation score, incorporating the evaluation of 10 distinct sets of cross-validation (Figure 6A). In the 10 cross-validations, the effect of the training and test sets became less effective as more tests were conducted, and the green line (lightGBM) outperformed all the other algorithms. Because of this, we ultimately chose lightGBM as the algorithm for the model. After choosing the LightGBM algorithm, hyperparameter training was performed. The input data were first divided into a test set and a training set, and then a search for hyperparameters was carried out. The appropriate parameters were selected using a plotted learning curve. By aggregating multiple hyperparameters, this model achieved an accuracy of 92.2%, representing an improvement of 13.68% over the lightGBM before tuning. To evaluate the efficacy of the model, a receiver operating characteristic (ROC) curve was constructed (Figure 6C). The closer the ROC curve is to the upper left corner, the better the performance of the classifier; the point on the ROC curve closest to the upper left corner is the best threshold for the lowest number of classification errors and the lowest number of false positives and false negatives. The ROC of the model is 0.97, which shows that the model has good prediction performance and is accurate and reliable and that there is no overfitting.

The mean absolute value of a feature’s degree of influence on the target variable was utilized to determine its significance. Based on the Shapley additive explanation (SHAP) values (Figure 6D), the importance of features depicts the mean absolute SHAP values to illustrate the importance of global features. The abundance of genes in the KEGG orthology gene function category provides insight into the genetic underpinnings of adaptive phenotypic variation. The bee swarm plot is intended to show a summary of how the top characteristics of a data set influence the model’s output. In each instance, the given explanation is represented by a single dot on each feature row. The x coordinate of the dot is determined by the SHAP value of that feature, and dots “pile up” along each feature row to represent density. Color is used to display the original value of a feature. Our investigation yielded insights into the genetic factors essential for the environmental adaptation of the yak gastrointestinal microbiota, highlighting the importance of the transcription-repair coupling factor (K03723), trk/ktr system potassium uptake protein (K03498), amino acid metabolism (K00817), and genes associated with polysaccharide and lipid metabolism (K05989, K01181). These identified genetic elements play pivotal roles in enabling the yak gut microbiota to effectively adapt to its surroundings. For example, the transcription-repair coupling factor likely contributes to genetic stability and maintenance in dynamic environments. Similarly, the trk/ktr system potassium uptake protein may support the maintenance of ionic balance critical for physiological functions. Amino acid metabolism, as well as polysaccharide and lipid metabolism genes, suggest the microbiota’s ability to efficiently extract nutrients from its environment.

Enrichment analysis based on these gene revealed that the microbial community of yaks exhibits more robust specific pathways compared to other plains ruminants (Supplementary Figure S1). These pathways encompass carbohydrate metabolism, nucleotide metabolism, transport systems, and amino acid metabolism, potentially playing a pivotal role in their adaptation and survival within their specific environment. The heightened activity of carbohydrate metabolism suggests efficient energy extraction from their diet, supporting energy-intensive processes. Emphasis on nucleotide metabolism might indicate enhanced DNA and RNA synthesis, potentially aiding cellular growth and repair. The well-developed transport system could facilitate nutrient absorption and intercellular communication, thereby optimizing resource utilization. Overall, these findings shed light on the mechanisms by which the yak gut microbiota adapts to its habitat and offers valuable insights into the genetic underpinnings of its environmental resilience.

## 4. Discussion

Ruminants are among the most successful herbivorous mammals (Decker et al., 2009). In this study, we took full advantage of the largest and most comprehensive database of ruminant gastrointestinal microbes available. We employed a range of approaches, encompassing complex networks and interpretable machine learning, to characterize the state of environmental microbial populations.

In this study, we employ network science theory to examine the properties of gastrointestinal networks and evaluate the robustness of these networks by examining these properties in more detail. Diverse profiles of topological features in diverse environmental networks reveal the unique co-occurrence patterns of microorganisms in ruminant species. The prevalence of cross-feeding relationships in the network may be indicated by the high clustering coefficient, which suggests that these settings have abundant degradation pathways, niche filtering, or environmental unpredictability. Different models built to test the resilience of networks reveal that they are more resilient to random faults and more vulnerable to deliberate attacks. Understanding the resilience of networks and the various approaches to averting catastrophic failures of these networks is essential. Only a tiny number of significant species have been eliminated, which may have an effect on the general structure of the network of microbes in a healthy microbiome. This finding highlights the importance of using antibiotics sparingly once more.

By investigating antibiotic resistance genes, we gain insights into how microbial communities respond to antibiotics. This understanding is pivotal, as it unravels the intricate interplay among microorganisms and is fundamental to comprehending the intricate web of interactions that shape ecosystems (Sharland et al., 2015). The presence of antibiotic-resistant microbiomes (ARBs) and ARGs in supermarket meat and dairy products suggests that ARBs/ARGs from ruminants can penetrate the food system. It would be helpful to make an effort to compile a list of significant ARG-carrying species for monitoring and control based on the assessment of the total antibiotic resistance risk at the species level. It is alarming that the farming environment contains high-risk MAGs. Additionally, microbiomes communities frequently experience an increase or decrease in ARGs as a result of genetic changes or HGT. Human health would be seriously endangered by these high-risk MAGs.

The Qinghai–Tibet Plateau, sometimes known as the “Third Pole,” is a huge, high-altitude region with a unique and fragile ecological environment (Yao et al., 2012). The region is characterized by a harsh climate of extreme cold, drought, high ultraviolet radiation and a lack of oxygen, making it a challenging living environment for humans and other mammals (Zhu et al., 2018; Pan et al., 2021; Shen et al., 2021). It is essential to determine precisely which genetic features give ruminants their exceptional digestive capacity and ability to live in harsh conditions (Zhu et al., 2020). To answer this question, we developed interpretable machine learning methods to deeply mine complex, high-dimensional metagenomic data. We found a significant increase in the transcription repair coupling factor (K03723) in the yak gastrointestinal microbiota. K03723 regulates transcriptional processes and recognizes DNA damage. In addition, such phenomena were also found in samples from plateau-based animals for *Rhodobacter* sp. (Pérez et al., 2018) and nitrogen-fixing microbiomes (Suyal et al., 2018). We hypothesize that this may be a common measure adopted by microorganisms facing extreme environments. In addition, we found that the K03498 trk/ktr system potassium uptake protein contributes significantly to plateau acclimatization. We speculate that the numerous high-salinity sites in the plateau region have resulted in a microbial response to salt stress (Li et al., 2021). Similar to previous studies (Guo et al., 2021),the results indicate a preference for an amino acid metabolism gene (K00817) and polysaccharide and lipid metabolism genes (K05989 and K01181) in the yak gastrointestinal microbiota. These pathways provide additional adaptive responses to the lack of energy intake in yaks. In conclusion, our study provides important insights into ruminant plateau adaptation and highlights the key role of the microbial genome as a “second genome” for adaptation, contributing to a more comprehensive understanding of mammals living in extreme environments.

## 5. Conclusion

In-depth exploration of ruminant gastrointestinal microbes is necessary to understand the function of the microbiome and its interactions with the host animal. This study enhances our comprehension of both the structure and function of the ruminant gastrointestinal microbiota, a critical aspect for investigating microbial-host symbiotic functional dynamics. Furthermore, it advances our understanding of the gastrointestinal microbiota adaptations necessary for herbivores. In addition, it informs strategies to decrease contamination and increase the robustness and efficiency of ruminants.

## 6. Methods

### 6.1. Data used in this study

The sequence files of 10,373 gastrointestinal microbial genomes of ruminants were downloaded in FASTA format from Figshare (DOI: 10.6084/m9.figshare.14176574). All the gene catalogs, annotation information, abundance profiles, assemblies, and predicted open reading frames (ORFs) from this study are available at https://microbiomejournal.biomedcentral.com/articles/10.1186/s40168-021-01078-x. The details of all samples used in this study are provided in Supplementary Table S1.

### 6.2. Network analysis

A Spearman correlation matrix was calculated based on the relative abundance of genera in each sample, and networks were graphed using Gephi (Bastian et al., 2009). Topological features were estimated with the igraph package (Csardi and Nepusz, 2006) (v1.4.1) in R 3.6.0.

The robustness of gastrointestinal networks can be regarded as the ability of the entire network to maintain the same performance when nodes in the network are affected by random factors and intentional damage. The survivability of networks can be measured by the change in network topological structure characteristics. We selected the relative size of the largest connected subgraph (RS) and relative connectivity efficiency (RE) after node failure as the measurement indexes of network survivability (Wei et al., 2021).


$$RS=\frac{N^{t}}{N}$$

where N is the number of nodes in the largest connected subgraph and N is the total number of nodes in the airline network. As the nodes in the network are attacked, the network splits into several subgraphs, and the relative size of the largest connected subgraph gradually decreases until the nodes are no longer connected with each other and finally become scattered nodes.

In this research, different attack strategies are developed to further understand the failure process of the network under different scenarios, specifically including random failures and targeted attacks (Wang et al., 2010). The strategies can be summarized as follows: First, there is the random attack strategy based on nodes. Second, the largest degree first attack strategy based on nodes. Third, the largest pagerank degree first attack strategy based on nodes.

## 7. Functional analysis of microbial genes

### 7.1. ARG annotation

The functional annotation of all identified proteins encoded by ARGs was based on sequence similarity searches carried out with DIAMOND BLASTP v2.0.9 (Buchfink et al., 2021) with the default settings against the HMD-ARG database (Li et al., 2021). ARGs were selected at a sequence similarity threshold of 75% and a score threshold of 60 (Yan et al., 2022). In our study, we utilized a set of equations to effectively quantify the antibiotic risk associated with the presence of ARGs within different Metagenome-MAGs in the context of the larger ecosystem. These equations, as described by equation (Zhang et al., 2022), were structured as follows:


$$\mathrm{Risk}=\frac{num:ARG}{\mathrm{total}:ARG}\times \frac{num:ARG:\mathrm{subtypes}}{\mathrm{total}:ARG:\mathrm{suntypes}}$$

In this context, “num_ARG” stands for the count of ARGs detected within a specific MAG, while “total_ARG” refers to the overall count of ARGs encompassing the entire ecosystem under consideration. On the other hand, “num_ARG_subtypes” represents the tally of distinct ARG subtypes found within the same MAG, and “total_ARG_subtypes” denotes the comprehensive count of distinct ARG subtypes present within the entire ecosystem. By applying this approach, we aimed to evaluate and quantify the potential antibiotic risk posed by the presence of ARGs within each MAG, within the broader context of the ecological system. This method allowed us to gain insights into the degree of antibiotic resistance-related risk associated with specific MAGs and their ARG compositions, contributing to a more comprehensive understanding of the ecosystem’s antibiotic resistance dynamics.

## 8. Machine learning model development

### 8.1. Data collection

In this experiment, 330 yak rumen microbial genomes were used as input data, along with 1992 other ruminant genomes. After comparing the protein sequences to the database using eggNOG, the proteins were grouped into different KOs (*KEGG Orthology*), with each cluster of KOs consisting of direct homologous sequences so that the function of the sequence could be inferred. The KO gene function matrix was built as an input file for machine learning.

### 8.2. Data preprocessing

Unbalanced data can pose challenges for machine learning models. Most machine learning models assume that the same number of samples is available for each class. Ignoring this problem can lead to errors in a few classes (and thus make the model sensitive to classification errors), causing ML models to ignore observations in a few classes. In the current work, the number of samples collected was uneven because the number of ruminant gastrointestinal samples varied from region to region. The synthetic minority oversampling technique (SMOTE) method was applied to overcome the adverse effect of learning data imbalance (Chawla et al., 2002).

### 8.3. Model development and tuning

Four machine learning models were developed to predict microbial plateau adaptive function in the Jupyter lab development environment (Perkel, 2018) using scikit-learn,^1^ Numpy (v1.15.3), Pandas (v0.23.4), Matplotlib (v3.0.1), and Scipy (v1.1.0) for experiments. The machine learning algorithms used for classification in this work were random forest (Qi, 2012), decision tree (Somvanshi et al., 2016), light gradient boosting machine (lightGBM) (Ke et al., 2017), and XGBoost (Chen and Guestrin, 2016). These are all integrated tree-based learning methods and are rated by the machine learning community as the most popular nonlinear models today. LightGBM is a fast and efficient GBDT algorithm in the open-source promotion framework designed by Microsoft MSRA in 2016. The algorithm is used for many machine learning tasks, such as sorting, classification, and regression, and supports efficient parallel training.

### 8.4. Shapley additive explanation

SHAP quantifies the importance of variables by leveraging Shapley values, a concept originating from cooperative game theory introduced by Shapley in (Bouneder et al., 2020). SHAP’s theoretical foundation is rooted in cooperative game theory, as highlighted by Lundberg and Lee in 2017 (Van den Broeck et al., 2022). The methodology explicates the model’s predictions by embracing the idea of additive feature attribution. The fundamental principle of SHAP is to decompose the explanation of a prediction into contributions from each feature (Wang et al., 2022). It assigns each feature’s contribution based on its Shapley value across different subsets of features, which is equivalent to a weighted average of feature contributions. Shapley values are a concept from cooperative game theory and denote the average contribution of a player across all possible coalition formations.

The SHAP value of feature i(ϕi) can be computed using the following equation:


$$\phi_{i}=\underset{S\subseteq N\backslash \left\{i\right\}}{\sum }\frac{|S|!\left(|M|-|S|-1\right)!}{|M|!}\left[f_{x}\left(S\cup \left\{i\right\}\right)-f_{x}\left(S\right)\right]$$

Where: N represents the set of all features. *S is a subset of NN that does not include feature i.* $f\left(S\right)$ represents the model’s prediction when considering only the feature set *S.* $f\left(S\cup \left\{i\right\}\right)$ is the model’s prediction when feature ii is added to the features in subset S. The idea behind this formula is to consider all possible combinations of features, excluding feature ii, and calculate the difference in model predictions when feature ii is included in these combinations. The Shapley value concept assigns weights to these differences based on the number of ways a specific feature can contribute to different subsets of features. The summation calculates the weighted average of these differences, yielding the SHAP value for feature *i.*

In essence, the SHAP value quantifies the contribution of each feature to the model’s prediction by considering how including or excluding that feature influences the model’s output across various combinations of features.

## Data availability statement

The original contributions presented in the study are included in the article/supplementary material, further inquiries can be directed to the corresponding author.

## Author contributions

YY conceived, designed the overall study, conceived, designed, and executed the bioinformatics analysis. XB and YG provided the genomic information. All authors contributed intellectually to the interpretation and presentation of the results in the manuscript, which was edited and approved by all authors.

## Conflict of interest

The authors declare that the research was conducted in the absence of any commercial or financial relationships that could be construed as a potential conflict of interest.

## Publisher’s note

All claims expressed in this article are solely those of the authors and do not necessarily represent those of their affiliated organizations, or those of the publisher, the editors and the reviewers. Any product that may be evaluated in this article, or claim that may be made by its manufacturer, is not guaranteed or endorsed by the publisher.

## Footnotes

^1^ https://scikit-learn.org
