# Supplementary figures and images for: Combined network analysis and interpretable machine learning reveals the environmental adaptations of more than 10,000 ruminant microbial genomes
Source: Front Microbiol. 2023 Sep 20;14:1147007. doi: 10.3389/fmicb.2023.1147007 (PMC10548237; doi:10.3389/fmicb.2023.1147007)

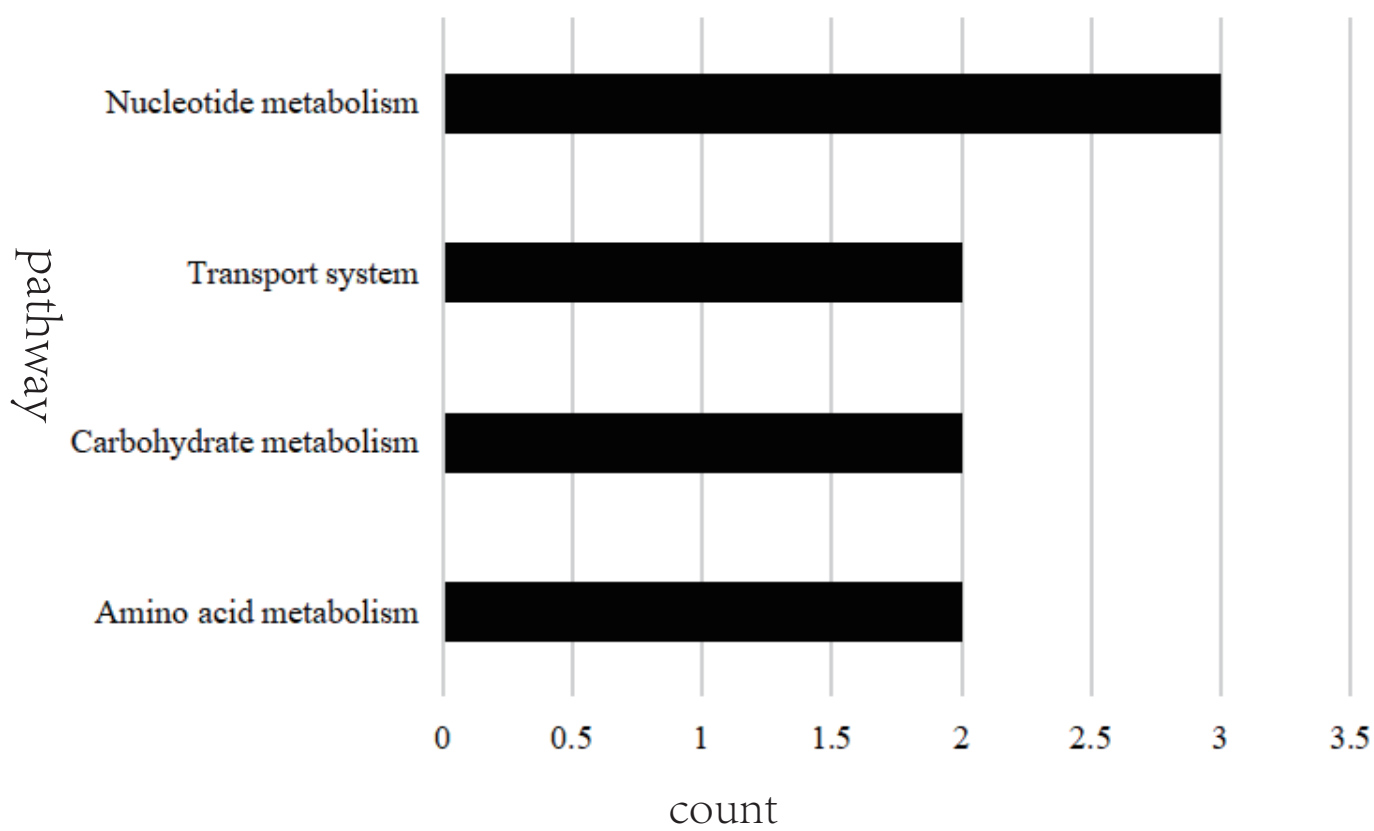

Supplement: Supplementary file 1 [file Image_1.pdf]
